# Fin whale (*Balaenoptera physalus*) migration in Australian waters using passive acoustic monitoring

**DOI:** 10.1038/s41598-019-45321-w

**Published:** 2019-06-20

**Authors:** Meghan G. Aulich, Robert D. McCauley, Benjamin J. Saunders, Miles J. G. Parsons

**Affiliations:** 1 Curtin University, School of Molecular and Life Sciences, Bentley, Western Australia 6102 Australia; 2 Curtin University, Centre for Marine Science and Technology, Bentley, Western Australia Australia; 3 Australian Institute of Marine Science, Fairway, Crawley, Western Australia 6009 Australia

**Keywords:** Animal migration, Behavioural ecology, Marine biology

## Abstract

The fin whale is a globally endangered species and is listed as threatened in Australia, however no peer-reviewed studies are available to indicate the migratory movements of the species in Australian waters. This study uses passive acoustic monitoring as a tool to identify the migratory movements of fin whales in Australian waters. Sampling was conducted from eight locations around Australia between 2009 and 2017, providing a total of 37 annual migratory records. Taken together, our observations provide evidence of fin whale migration through Australian waters, with earliest arrival of the animals recorded on the Western Australian coast, at Cape Leeuwin in April. The whales travel through Cape Leeuwin, migrating northward along the Western Australian coast to the Perth Canyon (May to October), which likely acts as a way-station for feeding. Some whales continue migrating as far north as Dampier (19°S). On Australia’s east coast, at Tuncurry, fin whale seasonal presence each year occurred later, from June to late September/October. A total of only 8,024 fin whale pulses were recorded on the east coast, compared to 177,328 pulses recorded at the Perth Canyon. We suggest these differences, as well as the spatial separation between coasts, provide preliminary evidence that the fin whales present on the east and west coasts constitute separate sub-populations.

## Introduction

The fin whale (*Balaenoptera physalus*) is the second largest baleen whale reaching 22 m in length and has a world-wide distribution^1^. Fin whales are currently listed as endangered under the IUCN Red List^2^ due to the steep population decline of global whale populations during the industrial whaling era^3^. Whilst the species is protected by global directives, they still face a wide range of threats to their recovery such as fisheries activity^4^, ship strikes^5^ and habitat disturbance^6^. Like other baleen whale species, fin whales are thought to conform to a stereotyped migration, with yearly seasonal movements from polar to sub-tropical/tropical waters for the winter months^7,8^. Due to the seasonal difference between the Northern and Southern Hemispheres, fin whale movements towards the equator are seasonally separated, as such there is no evidence of population overlap in the tropics^3,8^. Fin whales are therefore divided into Northern and Southern Hemisphere populations with two sub-species recognised; *B. physalus physalus* (Northern Hemisphere) and *B. physalus quoyi* (Southern Hemisphere)^9^. A sub-species of pygmy fin whale (*B*. *physalus patachonica)*^9^ is also present in the Southern Hemisphere^10^, however limited studies are available to validate and identify the species. Sub-populations of fin whales have also been recognised between different oceans and populations within oceans in the Northern Hemisphere^11–13^ as there is little evidence of mixing between the animals along different migratory routes. Understanding the migratory movements of the fin whale, and ascertaining potential sub-populations of the species is useful to aid in monitoring the recovery of this endangered species.

Passive Acoustic Monitoring (PAM), the recording of animal vocalisations, is a method often used to study marine mammals, that offers the advantage of sampling over long periods of time, in habitat and weather conditions that are not optimal for other methods of sampling (such as visual survey)^14^. PAM is a non-interactive technique that does not have any negative effect on whales or their behaviour^15^. The technique therefore provides a high temporal resolution of migration patterns, making it an ideal tool for sampling fin whale migration. To effectively use PAM for whale research, the acoustic repertoire and call characteristics of the species must be known. The fin whale has one of the most commonly known call types, with their vocalisations characterised by short, down-sweeping pulses^16–20^. The most widely reported and commonly produced are the “20 Hz” classic pulses which can be displayed in irregular pulse patterns or as repetitive pulse sequences, which can last up to 32 hours^17^. The individual pulses have a duration of ~1 s with intervals between pulses varying from 6–37 s^17^. Each pulse has a frequency bandwidth ranging from 13 to 40 Hz^21,22^, and is produced at a source level of approximately 189 dB re 1µPa^23,24^. These 20 Hz classic pulses have also been recorded accompanied by a higher frequency component ranging around 100 Hz^25^. A call display referred to as doublets has also been identified; doublets can be displayed in the form of “trains” with a repetitive sequence of two classic pulses at alternating intervals or as “back-beat” pulses whereby a lower-frequency pulse precedes a classic pulse, and are produced at alternating intervals^19,26–30^. A final call display, referred to as “40 Hz” pulses are characterised by higher frequency pulses, ranging from 100 to 50 Hz^16,25^. However, this call display is not widely recorded throughout the literature. Watkins *et al*.^17^ suggested that the 20 Hz repetitive sequence pulses made by fin whales are used as a breeding display, as fin whales adapted their call rates and characteristics in winter months, aligning with the copulation stage of their reproductive cycle^7^. Further studies have shown seasonal changes in call characteristics supporting this suggestion^18,19,27,29,31^, such as Širović *et al*.^32^ who observed a seasonality in the occurrence of the 20 Hz pulses. Croll *et al*.^33^ goes further to suggest that only male fin whales produce these call displays. There is however, a lack of replication of this finding within the literature, with no similar studies in the Southern Hemisphere. Our study therefore considers recorded fin whale vocalisations not as ‘male fin whale’ calls, but rather as ‘fin whale’ calls.

Using PAM, the migratory movements of Northern Hemisphere fin whale populations has been widely described, and their temporal and spatial presence in different regions defined^27,34–37^. PAM have also provided evidence of non-migratory, resident populations of fin whales recorded in the North Pacific^32,38^ and North Atlantic Oceans^31^. In addition to the migratory movements, acoustic studies can also give information on migratory routes. In the Northern Hemisphere acoustic recordings have demonstrated migratory winter presence of fin whales in the eastern and western regions of the North Pacific^32,39^ and the North Atlantic Oceans^31,40,41^. Taken together, these acoustical studies describe an aggregation of fin whales in polar waters in summer months, but different migration routes throughout the oceans for winter with some whales following a south easterly migration route, and others travelling south westerly. In contrast, movement patterns in much of the Southern Hemisphere are poorly understood. Acoustic studies of fin whales around the Antarctic continent suggest that seasonal movement is a slow dispersion of the animals out of Antarctic waters in the winter months and a return in the summer months^42^. Upon leaving Antarctica fin whales migrate into lower latitudes, with some moving to New Zealand^26^ and others as far north as Tonga^28^ for the winter months. However, no peer-reviewed studies are available to indicate the migratory movement of fin whales in Australian waters.

This study uses passive acoustic monitoring as a tool to identify fin whale seasonal presence around Australian waters. We aim to provide an overview of the seasonal migratory movements of fin whales around Australia. Specifically, this study aims to:

1. Identify non-migratory, resident populations,
2. Identify seasonal presence with timing of first arrival, and last vocalisations in Australian waters, and
3. Provide evidence for the migration routes the animals take within Australian waters.

## Results

**Figure 1:**
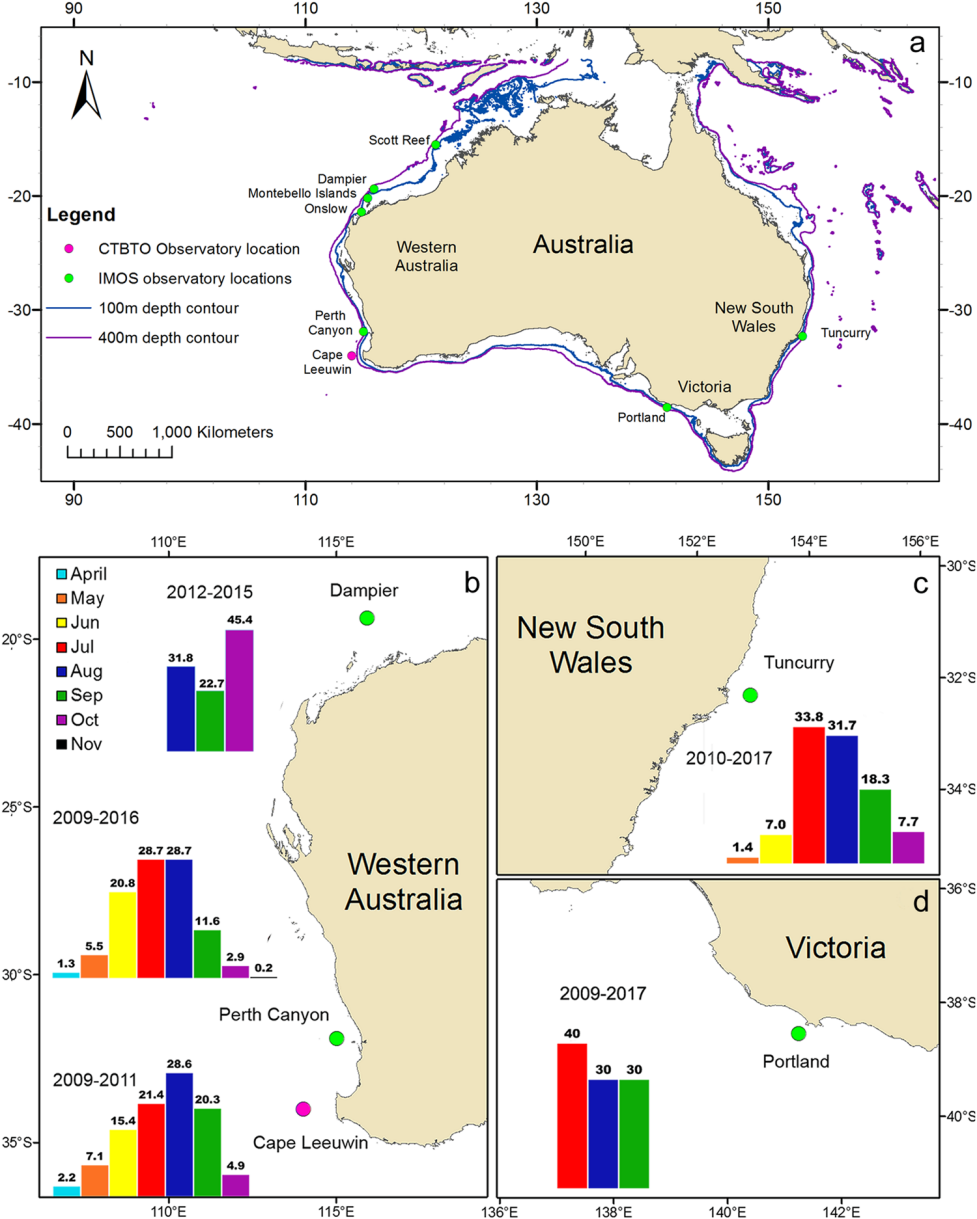
(**a**) Map of the locations of the passive acoustic Underwater Sound Recorders (USR) of the Australian Integrated Marine Observing System (IMOS) and Curtin University, and the Comprehensive Test Ban Treaty Organization (CTBTO) hydroacoustic array around Australian waters. And temporal pattern of fin whale presence around Australia, (**b**) off the Western Australian coast at Cape Leeuwin, the Perth Canyon and Dampier, (**c**) off the east coast at Tuncurry, NSW and (**d**) at Portland, Victoria. Bars represent the percentage of calling days for each month, all data sampling periods for each location were grouped.

**Figure 2:**
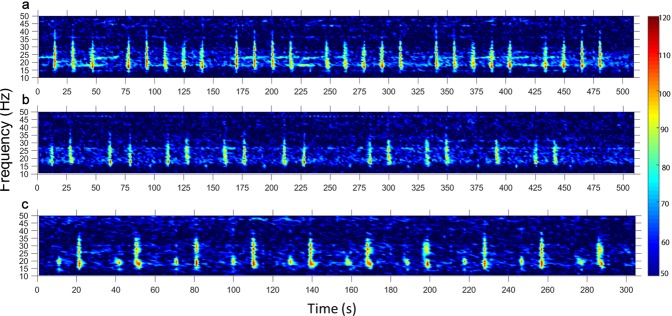
Spectrograms of an example of the three different pulse displays recorded by IMOS or Curtin USR’s: (**a**) the repeated pattern 20 Hz classic pulses (pygmy blue whale signals in the background), (**b**) the doublet train pulse display, and (**c**) the back-beat pulse display.

Using acoustic monitoring systems, a total of 247,294 fin whale vocalisations were recorded in Australian waters. Sampling was conducted from eight locations around Australia for one to nine years, between 2009 and 2017, providing a total of 37 annual migratory records (Fig. 1a.). No evidence of resident, non-migratory populations was found. These data provide an insight into the temporal migratory movements and routes undertaken by fin whales in the Southern Hemisphere. The 20 Hz pulses were recorded at all locations. Examples of call displays including the repeat pattern, stereotyped classic pulses, doublet trains and back-beat pulses are presented in Fig. 2. The 40 Hz pulses were not observed at any sites across all recording effort.

**Figure 3:**
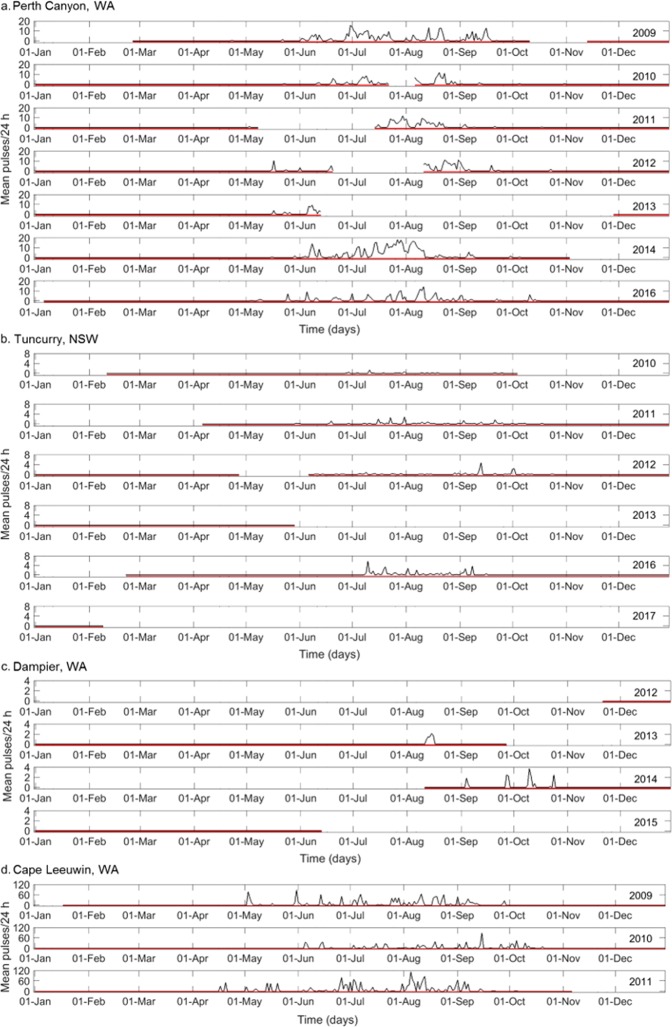
Mean number of fin whale pulses counted in every normalised 5 min sample averaged over 24 h (**a**) at the Perth Canyon, WA, (**b**) Tuncurry, NSW, (**c**) Dampier, WA. And the number of fin whale pulses recorded in every 1 h sample averaged over 24 h at (**d**) Cape Leeuwin, WA. Red horizontal lines indicate when recorders were deployed; no recordings were obtained during months without red lines. Note that the Y axis is different for each sampled location.

The Perth Canyon (Western Australia) and the New South Wales (NSW, eastern Australia) recording sites were at similar latitudes (≈ 32°S respectively), separated by the Australian mainland (≈ 2500 km or 26.24° of longitude). A comparison can therefore be drawn between them in regard to the time of arrival of fin whales in waters off Australia’s east and west coasts. These locations also had the most sampling effort, in terms of migrations observed. In the Perth Canyon recordings, the earliest detection of a vocalising fin whale within a year occurred on the 24^th^ of April, 2010 and the latest on the 1^st^ of November, 2010 (Fig. 3a). A pattern was identified amongst all sample years in the Perth Canyon, with a seasonal presence from May through to October (Fig. 3a). Fin whales at the Perth Canyon had a mean arrival date of the 10^th^ of May with a standard deviation of 5 days (n = 7). The Perth Canyon had the greatest number of fin whale vocalisations recorded across all sites (177,328 pulses), with a high of 58,045 individual pulses recorded over the year 2009. The peak 24 h calling period in the Perth Canyon varied from year-to-year, with the maxima 24 h mean of 18.7 fin whale pulses/day occurring in late July, 2014 (Fig. 3a).

Off Tuncurry, NSW a different pattern of presence was found, with presence from June to late September/October, and with the first detection in a year not observed until the 30^th^ of May (2011). The latest detection across all years was on the 23^rd^ of October (2012), with no presence recorded into November in any year (Fig. 3b). An anomaly was observed in the 2016 season with the earliest fin whales being recorded late, on the 7^th^ of July and the last call early, on the 15^th^ September, 2016 (Fig. 3b). Mean arrival time of fin whales at Tuncurry was significantly later than at the Perth Canyon (t_(9)_ = −4.75, p = 0.001). At Tuncurry the mean arrival date was the 19^th^ of June, with a standard deviation of 7 days (n = 4). A total of 8,024 individual pulses were recorded at Tuncurry across all data years, with the greatest number recorded in 2016 (3,937 pulses). Peak calling periods were less defined in Tuncurry, with several shorter peaks within months. The maxima 24 h mean of 5.8 fin whale pulses/day occurred in early July, 2016 (Fig. 3b).

Passive acoustic systems were deployed at four northerly locations on the Western Australian coast. However, no fin whale vocalisations were detected at three of these locations. None were recorded at Scott Reef (15.29°S) throughout three years of surveying, (total of 6,703 hours of recording collected), Onslow throughout two years of surveying (total of 1,850 hours of collected recording), or Montebello Islands across one year of surveying, (1,175 total hours of recording collected). At the Dampier location (19.23°S), fin whale singing presence was recorded in two survey years. At Dampier, the earliest detection of fin whales within a year occurred on the 13^th^ of August 2013 and the latest on the 25^th^ of October 2014 (Fig. 3c). A total of 749 fin whale pulses were recorded in 2013 and 1,705 in 2014. The maxima 24 h mean of 3.6 fin whale pulses/day occurred in mid-October, 2014.

Approximately 26,280 hours of continuous recording were collected at Cape Leeuwin, WA from 2009–2011, with a total of 59,098 pulses throughout the entire recording period. Due to the continuous recording scheme of the CTBTO hydroacoustic station, this is the most accurate representation of daily fin whale vocalisations. The earliest detection of a vocalising fin whale within a year at Cape Leeuwin occurred on the 18^th^ of April, 2011 and the latest on the 21^st^ of October, 2010 (Fig. 3d). A peak in calling periods was identified with the greatest mean number of pulses per day occurring in late June, 2009 (87.6 fin whale pulses/day), mid-September, 2010 (89.5 fin whale pulses/day) and August, 2011 (112.5 fin whale pulses/day) (Fig. 3d). Seasonal presence of fin whales show yearly variation, and as Cape Leeuwin has samples overlapping with recordings from the Perth Canyon, comparison can be made between them in regard to arrival and departure times. In general, the first and last arrivals each year were recorded at the Perth Canyon (Fig. 3a) even though it was further north than Cape Leeuwin. For example, in 2009, fin whale presence was recorded first at the Perth Canyon (24^th^ of April) and 10 days later at Cape Leeuwin (4^th^ of May). The only exception to this occurred in 2011 with first fin whale presence recorded at Cape Leeuwin on the 18^th^ of April, and in the Perth Canyon 15 days later on the 3^rd^ of May.

**Figure 4:**
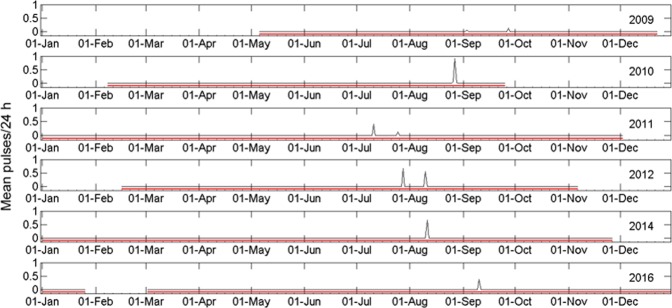
Mean number of fin whale pulses counted in every 5 min sample averaged over 24 h at Portland, VIC. Only sample years with detected fin whale signals are presented. Red horizontal lines indicate when recorders were deployed; no recordings were obtained during months without red lines.

The recorded fin whale presence at Portland, VIC (Victoria, eastern Australia) was anomalous in comparison to all other locations. A total of only six hours with fin whale calling was recorded out of nine years of deployment, or a total of 22,670 hours of recording time collected. Only 390 fin whale pulses were recorded. The detections at Portland showed yearly variations, with the earliest calls detected on the 11^th^ of July, and others in late July, mid-to-late August and early and late September (Fig. 4). Calling was irregular, with some animals only calling for a few hours, or with calls at the start of a month and then several weeks’ silence before the next call presence. No vocalising animals were present during the 2015 survey period.

Together, these observations provide evidence of a pattern of fin whale migration in Australian waters (Fig. 1b–d), with first arrival in April at the Perth Canyon and Cape Leeuwin. Fin whales reside at the Perth Canyon between May and late October. Some whales continue as far north as Dampier where they were present between August and October. The last detections of whales on the return journey were at Cape Leeuwin in late October (Fig. 1b). On the east Australian coast at Tuncurry, NSW fin whales have a seasonal pattern of arrival in June and the animals leave in late September/October (Fig. 1c).

## Discussion

Our study provides the first systematic assessment of the seasonal migratory movements of vocalising fin whales in Australian waters. No vocalisations were observed year round at any sites, thus there is no evidence of non-migratory, resident populations of fin whales in Australia, in contrast to those found in the Northern Hemisphere^31,32,38^. Our data outlines clear seasonal patterns in calling presence, indicating the presence of fin whales in Australian waters as a winter migration. It is important to note that while a call indicates the presence of the whale, a lack of calls does not confirm an absence of whales, rather that there are no vocalising animals or that no whales have been detected by the acoustic monitoring systems.

Our study aimed to identify not only temporal presence of fin whales, but to also elucidate potential migratory routes of the animals through Australian waters. Other migratory baleen whale species, such as the humpback whale (*Megaptera novaeangliae*) exhibit fidelity to migration patterns in Australian waters, commonly migrating in large numbers as far north as 18–12°S, with different regions of coastal waters for specific habitat use, such as calving in the Western Australian Kimberly region or nursing grounds in the Exmouth Gulf^43^. Our study outlines a pattern of fin whale movement into Australian waters, appearing first off the Western Australian coast at Cape Leeuwin in April and lastly in October, before returning to Antarctica. The intermittent vocal presence of fin whales at Cape Leeuwin from April to June aligns with suggestions of fin whale migration as a slow dispersal of the animals out of Antarctic waters, rather than a mass movement^42,44^. This trend leads us to suggest that Cape Leeuwin is a travelling zone, with the animals passing through this region. The relatively low number of fin whale pulses at Cape Leeuwin from April to June may be because not all migrating fin whales are vocally active whilst travelling, or not all vocalising animals are within a range where they will be detected. Therefore, true first presence of the animals in Australian waters is likely to be earlier than reported here. Alternatively, some migratory fin whales may be approaching from the South West, directly to the Perth Canyon, not travelling past Cape Leeuwin. The Perth Canyon appears to be a consistently used habitat over a long-term period with presence occurring over seven consecutive years. This site also exhibited the longest seasonal presence (May to October) and the greatest number of vocalisations of all Australian sites analysed. The Perth Canyon’s physical properties promote aggregation of zooplankton in the canyon^45^, making it an attractive feeding area for migrating whale species such as the pygmy blue whale (*Balaenoptera musculus brevicauda*). We suggest that in a similar way, the Perth Canyon is likely to be a way-station for migrating fin whales for the purpose of feeding.

With no fin whales recorded at our most northerly site, Scott Reef (15.29°S), Dampier is our most northerly location (19°S) with fin whale detections. An acoustic study carried out in waters around Tonga reported migratory presence of fin whales in latitudes of 22–18°S^28^, so the detection of fin whales as far north as Dampier is not unexpected. The difference between the number of whale vocalisations in the Perth Canyon and Dampier datasets could be due to a wider spread and less dense population of fin whales in the north compared to the dense population migrating through the Perth Canyon. Alternatively it could be an indication that fewer animals travel as far north as Dampier. On their southward journey, the pattern of dense calling rates of fin whales in August at Cape Leeuwin can be explained by a mass movement of the whales in this region, as they migrate out of Australian waters. Alternatively, it could stem from the whales lingering at this location before their southward journey back to Antarctica. Fin whales migrating as far north as Dampier are travelling an annual migratory distance of approximately 10,000 to 11,000 km return from Antarctic waters.

On Australia’s east coast, the sporadic calling times and the small number of fin whale calls recorded in Portland, VIC indicate an inconsistent and irregular presence of fin whales in this region of the southern Australian continental shelf. This suggests that Portland is not frequented by fin whales and may not be part of a defined migratory route for the animals. Vocal detections at Portland in July might come from animals migrating northward before reaching Tuncurry, and presence of the animals in late August and September are likely from fin whales moving southward on their migration back to Antarctic waters. Analysis of the call characteristics of the singing whales at this location and how they differ or replicate other locations could help to confirm migratory patterns and the habitat use of this area. The overall temporal pattern of fin whale calling presence observed at the Tuncurry location, NSW (June to September/October) aligns with the previously observed migratory movements of fin whales in the South Pacific, in regions of New Zealand and Tonga (June to October)^26,28^. This pattern of movement differs from that observed on the Western Australian coast, where we found a pattern of earlier arrival time and therefore longer calling presence (May to October). This difference in migration timing between coasts could be due to fin whales leaving Antarctic waters at different times, reflecting variation in the timing of annual sea ice formation in different regions of Antarctica. Fin whales migrate away from polar regions when sea ice increases^22,36,44,46,47^. The origin of the migrating animal could also affect the travel and arrival time of the whales. Those whales coming from more distant regions in Antarctica^42^ may have a greater distance to travel before arriving in waters off the Australian mainland. Alternatively, the animals may aggregate in regions south of Tuncurry for periods of time before travelling further north. Future acoustic monitoring with receiver placement along Australia’s east coast would provide more information into the migratory movement of east coast fin whales.

The Perth Canyon and NSW recordings each displayed seasonal patterns in peak calling. The peaks in whale calls indicated an overall increase through the year, which could indicate a gradual increase in the number of animals present, as more animals arrive at each location. However, the possibility that this pattern could instead be due to an increase in calling rate cannot be ignored. If it is due to an increase in animal numbers then this also supports suggestions that fin whale migration is a slow dispersion out of Antarctic waters^42,44^.

In all years analysed, a greater number of fin whale vocalisations were recorded on the Western Australian coast than on the east coast. This difference in number of vocalisations could indicate presence of a greater number of vocalising fin whales on the Western Australian coast than on the east coast. The difference in fin whale numbers between coasts could stem from a range of environmental factors such as habitat type preference^48^ or prey preferences and/or prey abundance^49^. This may encourage longer residence time for individuals in the Perth Canyon compared to those passing the shelf break off Tuncurry in the east. The origin of the animals’ migratory journey from Antarctic waters could also influence the migration destination. Fin whales originating from different regions of Antarctica i.e. East or West Antarctica^42^, may undertake easterly or westerly migrations respectively. We suggest that the spatial separation, difference in seasonal presence and the difference in the number of whale vocalisations between coasts provide evidence that these are two different sub-populations of fin whales.

Signal detection ranges in passive acoustic monitoring are source-, site-, and condition- specific and therefore highly variable. Factors pertaining to the source signal, such as call frequency range, call source level and the position and depth of the animal relative to the receiver affect signal propagation and therefore detection range^50^. The bathymetry, seafloor geology and vertical sound speed profile (salinity and temperature with depth) all affect transmission losses across a particular area^51,52^, thus the detection range of a receiver is not spherically uniform, rather, dependent on the bathymetry and water column conditions in different headings from the receiver^50^. Background noise at the receiver, such as other calling whale species or man-made signals such as noise generated by ships or petroleum drilling, all impact the signal-to-noise ratio, therefore reducing detection range to different extents^51^. Similar receivers to those used in our study have been shown to have a detection range for fin whales of between 30 and 190 km, depending on these factors^50^. Therefore it is possible that whales were present nearby, and possibly calling, at times other than we report here, but that they could not be detected.

While detection range brackets for this study have not been calculated, broad statements can be made based on the likely sound transmission environment at each location. Sites where the receiver was placed in deep water and/or along the continental shelf i.e. Tuncurry (NSW), Dampier (WA), Cape Leeuwin (WA), Perth Canyon (WA), and Portland (VIC) would have a greater detection range with further range offshore (likely near 100 kms) than inshore (likely several 10’s kms), due to the increasing depth of the shelf. In comparison, the receivers at Onslow and Montebello Islands would have limited detection ranges (10 s kms only) due to the shallower environment, but still were capable of detections in offshore waters. While fin whales have been described as an offshore species^8,53^, acoustic studies have also recorded presence of the animals inshore of the continental shelf, within 10 km of land^31,32,38^, and sightings of the animals at depths as shallow as <40 m^54^. Despite these potential limitations, the placement of the recorders used in this study was designed to maximise likelihood of detection of whales in both inshore and offshore zones.

The lack of fin whale call detections at Onslow and Montebello Islands could stem from a range of factors. The limited detection range of acoustic receivers at these two locations, due to the sound transmission environment (shallow water), is likely the most important contributor to this. Other factors, such as anthropogenic activity, which varies from year to year in the region^55,56^, could act as a deterrent, as fin whales are known to alter their behaviour in response to anthropogenic noise^6^. Additionally, habitat preference of individual whales may contribute, with shallow reef habitats such as at these locations^55^ being less suitable than deep water habitats. Alternatively, the lack of detections may reflect similar observations from the Mediterranean Sea, where fin whales do not follow a traditional migration route, rather nomadic opportunistic dispersion based upon prey availability^57,58^. Further long-term passive acoustic studies with broader grid-like system placement, would fill in the spatial and temporal gaps, allowing for greater detection coverage in these regions, and could further clarify the migratory route of fin whales around these locations. In addition, future studies could seek the origin of the migrating animals, and track their movements out of Antarctic waters.

This study provides key information for the future management of this globally endangered species^2^. They are listed as threatened under the Australian EPBC act^59^, and endangered in WA. Due to a lack of data there is no current listing in NSW or Victoria^59^. By analysing a total of 37 annual migratory records, obtained from eight sampled locations around Australia, our study has identified a winter migration of Southern Hemisphere fin whales into Australian waters from April to November, with no evidence of resident, non-migratory populations. A significant difference in migration timing, the number of whale calls recorded between the east and west coasts, and the spatial separation between coasts leads us to suggest that the fin whales on these different coasts constitute different sub-populations. Further studies combining our long term data analysis with analysis of the call characteristics of the vocalising fin whales from Australia and Antarctica could help to define these populations and increase our knowledge of habitat use by the animals while in Australian waters. This provides the first systematic study of the northward winter migration of Southern Hemisphere fin whales into warmer waters, which is useful for the future management of this globally important species.

## Methods

### Data acquisition

The data that support the findings of this study are available from the Integrated Marine Observing System (IMOS) (http://imos.org.au/data/) and the Comprehensive Test Ban Treaty Organization (CTBTO) preparatory commission (https://www.ctbto.org/verification-regime/the-international-data-centre/distribution-of-data-and-data-bulletins-to-member-states/).

To obtain long-term recordings of fin whale sounds, data were taken from two separate acoustic monitoring systems:

1. Underwater Sound Recorders (USR) comprised an autonomous underwater sound recorder calibrated at 2 Hz to 2.8 kHz (usable bandwidth, sample rate 6 kHz), a hydrophone, preamplifier, flotation, stabilising bars, and batteries capable of long-term (10–12 month) deployment^60^. Data were sampled by observatories with a pre-set recording scheme varying from 5 or 7.5 minutes every 15 minutes at a sample rate of 6 kHz. Due to the varying deployment periods, not all samples cover the whole migration period. USR’s of the Australian Integrated Marine Observing System (IMOS) or collected by Curtin University, were deployed at seven locations around Australia between 2009 and 2017 (Fig. 1a). The best spatial coverage of observatories was off Western Australia (WA) with five stations, while on the east coast of Australia there were two observatory locations, one station in Victoria (VIC) and another in New South Wales (NSW) (Fig. 1a). Recording effort varied over time, with the longest coverage of nine years in Victoria, and the shortest coverage of one year at Montebello Islands, WA. The longest consecutive recording time of 386 days occurred in NSW from 2011–2012 (Table 1).

**Table 1:** Details of deployments of the passive acoustic USR of the Australian Integrated Marine Observing System (IMOS), Curtin University and the CTBTO station HA01 off Cape Leeuwin. The positions, start and end times, sampling days and approximate depths and distance from shore are provided for each deployment.

Location & set	Latitude	Longitude	Start time	End time	Days	Depth (m)	Distance from shore (km)	Total Pulses
**Perth Canyon, WA**								
2823	31 54.466	114 59.080	25-Feb-2009	12-Oct-2009	229	465	50	**177,328**
2884	31 55.039	115 1.863	13-Nov-2009	22-Jul-2010	251	—	—	
2962	31 54.139	115 1.607	06-Aug-2010	08-May-2011	275	—	—	
3004	31 54.350	115 1.538	14-Jul-2011	20-Jun-2012	341	—	—	
3154	31 53.053	115 0.813	10-Aug-2012	14-Jun-2013	307	—	—	
3376	31 50.530	115 0.824	28-Nov-2013	04-Nov-2014	340	—	—	
3445	31 52.656	115 0.656	17-Dec-2015	30-Dec-2016	380	—	—	
**Onslow, WA**								
2842	21 25.00	114 49.960	22-Jul-2009	04-Nov-2009	106	45	36	**0**
2925	21 45.969	114 49.924	26-Mar-2010	04-Nov-2010	223	—	—	
**Montebello Islands, WA**								
2919	20 11.284	115 23.566	24-Mar-2010	26-Oct-2010	216	45	22	**0**
**Dampier, WA**								
3188	19 23.291	115 54.896	20-Nov-2012	27-Sep-2013	216	216	150	**749**
3334	19 22.514	115 56.054	11-Aug-2014	13-Jun-2015	205	—	—	
**Scott Reef, WA**								
3187	15 29.002	121 15.056	20-Nov-2012	29-Sep-2013	216	216	200	**0**
3250	15 29.002	121 15.060	01-Oct-2013	02-Jun-2014	216	—	—	
3335	15 29.000	121 15.060	19-Aug-2014	08-May-2015	216	—	—	
**Cape Leeuwin, WA**								
	34 0.89	114 0.13	01-Jan-2009	31-Dec-2011	1,094	1050	90	**59,098**
**Portland, VIC**								
2846	38 32.981	141 15.235	06-May-2009	22-Dec-2009	229	168	10	**390**
2926	38 33.031	141 15.232	07-Feb-2010	25-Sep-2010	229	—	—	
3102	38 33.604	141 15.125	30-Dec-2010	03-Dec-2011	338	—	—	
3073	38 32.559	141 13.047	15-Feb-2012	06-Nov-2012	264	—	—	
3184	38 32.034	141 14.589	07-Nov-2012	17-May-2013	190	—	—	
3274	38 32.218	141 14.854	30-Dec-2013	27-Nov-2014	331	—	—	
3381	38 32.521	141 13.263	03-Feb-2015	26-Jan-2016	357	—	—	
3446	38 32.749	141 13.269	01-Mar-2016	21-Feb-2017	357	—	—	
**Tuncurry, NSW**								
2947	32 19.362	152 56.672	10-Feb-2010	04-Oct-2010	236	190	34	**8,024**
3142	32 19.128	152 56.721	06-Apr-2011	26-Apr-2012	386	—	—	
3128	32 17.396	152 54.612	05-Jun-2012	30-May-2013	358	—	—	
3428	32 18.590	152 55.839	21-Feb-2016	08-Feb-2017	353	—	—
2. The Comprehensive Test Ban Treaty Organization (CTBTO) nuclear test monitoring station operates an acoustic monitoring system south-west of Cape Leeuwin, WA (site HA01, Table 1, Fig. 1a). The system comprises three acoustic receivers suspended from the seafloor, with cables transmitting signals back to a facility on shore. Recording is continuous, with only short gaps. The hydrophones have a sample rate of 250 Hz. The data from a single hydrophone in this array was analysed for the years 2009 to 2011 (Table 1). The data was sourced from the CTBTO through Geoscience Australia, the Australian operator of station HA01.

### Call detections

**Figure 5:**
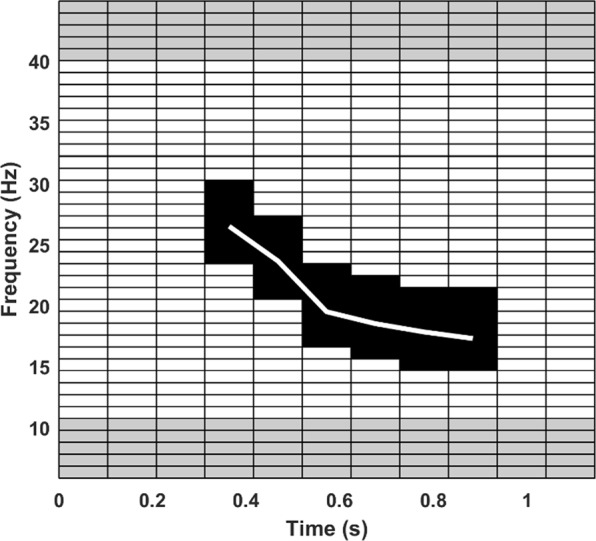
Image of template used in spectrogram cross correlation in the first algorithm. The white line is the frequency trend of a high SNR call, the black values are the padded spectrogram template area and the grey areas are the locations used for comparing noise levels above and below the fin whale pulse frequency band (14.6 Hz, to 29.3 Hz).

Fin whale calls were detected using two detection algorithms implemented in MATLAB, where a detected call was classed as a ‘hit’ with an allocated time stamp (time at which maximum spectral intensity within the pulse was reached). An algorithm checked a full ‘sample’ of 5 to 7.5 minutes (non CTBTO samples) or 1 hour (CTBTO samples) of sea noise for the presence of fin whale pulses, returning ‘hits’ with the pulse start time within the sample and various parameters for signals deemed to be fin whale pulses. The first algorithm was used to locate ‘samples’ with fin whale pulses. The presence of fin whales in these samples was verified by manual checking and the output of this manual checking ‘bracketed’ to check surrounding samples for missed detections. Then a second, more sensitive algorithm was run across samples with verified fin whale pulses, to locate individual pulses within a sample. The first algorithm worked by:

1. Down sampling the input signal to 500 Hz and calibrating it to units of Pa;
2. Checking for presence of high energy (>4 dB above ambient levels in surrounding bandwidth) spectral peaks occurring in the fin whale bandwidth (15–40 Hz);
3. Using a normalised, synthesised ‘fin whale’ pulse (single pulse at 18 Hz with exponential rise and decay of correct length), to cross correlate the input sample with the template and accept periods where the maximum squared cross correlation value was >500–1000 times the median ‘noise’ in the cross correlation (noise calculated as median of lower 1/3 of cross correlation values), accepting sections where this was true and using the time of cross correlation peak as the time of maximum energy in the pulse;
4. Setting a ‘window’ for potential hits as 0.5 s before and 2.2 s after the time for a hit given by 3), and retrieving the waveform and spectrogram of each of these sections (sample-spectrogram, 512 point FFT, 0.98 Hz resolution, 0.9 overlap Hanning window for a 0.10 s time resolution). These before and after times were selected as the highest level fin whale pulse energy arrived early in the pulse and sufficient time after was required to fully capture an entire pulse down sweep but which was not too long so as to impinge on a possible following pulse;
5. For a high signal to noise ratio, ‘20 Hz’ fin whale pulse, tracking the spectral energy across the call to produce a spectral line with time at the same resolution as the sample spectrogram, padding this line by ± 3 frequency points and building an index of these points into the sample spectrogram matrix. This was only done once. For the sample-spectrogram the mean noise level was found from the values in the fin-whale pulse bandwidth at the start and end of the window extracted. This mean noise value was subtracted from the points in the sample spectrogram falling within the index derived from the high SNR fin whale pulse above. This checked that the signal had consistent energy following the fin whale pulse, with the expected frequency decline with time. The call was accepted as a potential ‘hit’ if; (a) the number of values on the sample-spectrogram in the period defined by the high SNR call template index which were >3 dB above the pre and -post noise, was >30% of the total number of values in the fin whale spectrogram template; (b) the mean squared noise levels for 0.5 s before, and 0.5 s after the ‘window’ extracted were 3 dB less than the mean squared pressure across the ‘window’; and (c) the relative noise in the frequency band 5.9–9.8 Hz (below the fin whale pulse) and 40.0–44.9-(above the fin whale pulse) were >3 dB below the level measured in the template or ‘window’ area bound by 14.6 to 29.3 Hz, or in the fin whale pulse, energy band. An example of the spectrogram template, showing the areas used for comparing noise levels above and below the pulse with noise in the pulse frequency band, is shown in Fig. 5.

Once the first detection algorithm had been applied, the samples (in 5–7.5 minute sections) with ‘hits’ were displayed as spectrograms, where samples were manually viewed and interrogated. A sample spectrogram was deemed to either contain fin whale pulses (accept) or to not contain them (reject). For efficiency, the system only loaded samples which had not been previously manually checked and which showed detections according to the search algorithm. Once detections of all samples containing ‘hits’ as given by the first search algorithm had been confirmed, the program returned blocks of three samples before and after each sample containing a manually verified ‘hit’ and set this for checking, again only displaying samples not previously checked. This process was iterated until all three samples surrounding validated or newly selected ‘hits’ had been deemed to not contain fin whale pulses. A second, more sensitive detection algorithm was then implemented across samples with confirmed ‘hits’, due to low SNR calls that were not detected by the first algorithm. Manual checking of all data was not undertaken after this step except for an error determination process, due to the large number of fin whale pulses and size of data sets. This second algorithm only looked across samples in which ‘hits’ had been detected from the above detection and verification process. The second algorithm looked for signals matching a specified shape and length and worked by:

1. A high SNR fin whale call was loaded, down sampled to 250 Hz, and the absolute value of the Hilbert Transfer of the call found and normalised (0–1) to give a normalised template of the call envelope (curve of time and normalised intensity);
2. The input sample signal was down sampled to 250 Hz and band pass filtered (allowing energy in 8–45 Hz band);
3. The absolute value of the Hilbert transfer of the sample signal (positive signal envelope) was calculated, smoothed (running linear fit, 125 points either side);
4. The sample waveform and smoothed Hilbert transfer were split into 8.19 s windows, (2048 points) with each window having a 0.9 overlap with adjacent windows (giving curves of time with the normalised signal envelope for each window);
5. The squared error of the normalised template envelope minus the normalised, smoothed envelope for each window was found. Templates which matched the window curve thus gave low squared error values, those curve sets which were a poor match gave high values;
6. A binary noise rejection was set, with a value of one when the mean spectral noise levels above (35–40 Hz) and below (10–15 Hz) the dominant fin whale call frequency band (18–25 Hz) in the window analysed were >3 dB below energy in the call frequency band;
7. Any window where the inverse of the summed error of the template and sample window was above a threshold (20) and the criteria for noise rejection passed, was considered as a ‘hit’.

The final detection output (all of above steps) yielded a false detection rate of <1%, and a miss-detection rate of <4% for individual fin whale pulses. These misclassification rates were calculated by analysing a randomised selection of one thousand samples (five minutes each sample) containing fin whale hits from data recorded in the Perth Canyon in 2009 (set 2823). This data set was chosen as it had the greatest confounding noise, such as air-gun signals and shipping activity.

Counts of fin whale pulses from the IMOS acoustic observatories (pre-set recording scheme varying from 5 or 7.5 minutes every 15 minutes) were normalised to a 5 min sample length, CTBTO data (continuous recording) were left as 1 h long samples. All data from each recording site and recording period were averaged over 24 h periods to plot relative changes in fin whale vocalisation detections over time periods of greater than one day and limit any effects of diel patterns in calling behaviour on these patterns. An independent T-test at α = 0.05 was used to analyse statistical differences in mean (±SD) arrival times of fin whales between the Perth Canyon and Tuncurry locations. A pattern of fin whale calling presence at each location was calculated using the percentage of days within each month where at least one fin whale signal was detected. These proportions were illustrated and overlaid on a location map. For these calculations all data sampling periods for each location were combined in order to represent the seasonality of fin whale vocal presence around Australia.
